# Electrospinning With Lyophilized Platelet-Rich Fibrin Has the Potential to Enhance the Proliferation and Osteogenesis of MC3T3-E1 Cells

**DOI:** 10.3389/fbioe.2020.595579

**Published:** 2020-12-01

**Authors:** Jing Nie, Shumei Zhang, Peng Wu, Yuangang Liu, Yanjun Su

**Affiliations:** ^1^Stomatological Hospital of Xiamen Medical College, Xiamen, China; ^2^Xiamen Key Laboratory of Stomatological Disease Diagnosis and Treatment, Xiamen, China; ^3^Engineering Research Center for Stomatological Biomaterials, Xiamen Medical College, Xiamen, China; ^4^Technology Innovation Center for Exploitation of Marine Biological Resources, Third Institute of Oceanography, Ministry of Natural Resources, Xiamen, China; ^5^College of Chemical Engineering, Huaqiao University, Xiamen, China

**Keywords:** lyophilized platelet-rich fibrin, electrospinning, osteogenesis, cell proliferation, MEC3T3-E1

## Abstract

Platelet-rich fibrin (PRF) as a reservoir of various growth factors plays an essential role in wound healing and tissue engineering at present. Electrospinning technology is an efficient approach to acquire artificial scaffold which has large specific surface area and high porosity. The goal of this study was to investigate the potential of electrospinning on the proliferation and osteogenesis of osteogenic precursor cells *in vitro*, with lyophilized PRF added as a component for electrospinning preparation. The surface structure of lyophilized PRF and nanofibers were investigated, and the proliferation, osteogenesis of MEC3T3-E1 cells with lyophilized PRF or nanofibers extract were studied. The results showed that the diameters of the lyophilized PRF pores were 1.51 ± 0.75 μm, and lyophilized PRF medium promoted the proliferation and osteocalcin (OCN) and osteopontin (OPN) genes expression of MEC3T3-E1 cells. Furthermore, the diameters of the polyvinyl alcohol/sodium alginate/lyophilized PRF (PVA/SA/PRF) fibers were 201.14 ± 40.14 nm. Compared to PVA/SA nanofibers extract and control medium, PVA/SA/PRF nanofibers extract also enhanced the proliferation and mineralization activity of MEC3T3-E1 cells. These results might be instructive to future therapeutics with PVA/SA/PRF electrospinning for bone tissue engineering or other applications.

## Introduction

Platelets are rich in various bioactive factors and present excellent potential in tissue regeneration. Platelet-rich fibrin (PRF) is a second-generation platelet concentrate to replace platelet-rich plasma (PRP), and has become an attractive strategy in tissue grafting and regenerative medicine because it has abundant tissue healing-promoting cytokines and interleukins (ILs), including transforming growth factor beta 1 (TGF-β1), basic fibroblast growth factor (bFGF), platelet derived growth factor (PDGF), vascular endothelial growth factor (VEGF), IL-6, IL-8, IL-11, and so on (Masuki et al., 2016). Furthermore, PRF has been verified to increase tissue retention, quality, and neovascularized capillary density of grafted fat (Xiong et al., 2019). In addition, PRF has also been demonstrated to facilitate the regeneration of bone, periodontal tissue, and dental pulp (Chang and Zhao, 2011; Ji et al., 2015; You et al., 2019).

As the second-generation platelet concentrate, PRF is considered as an outstanding natural biomaterial scaffold based on fibrin. However, either used as a scaffold for tissue engineering or as a medical dressing for clinical application, fresh PRF encountered some limitations for its further application. For instance, it is not suitable for storage or transit in liquid nitrogen or on dry ice (Morris, 2005). Therefore, previous studies underline the preparation for immediate use (Choukroun et al., 2006). Furthermore, the gelatinous morphology of the fresh PRF lacks stable shape and resistance to compression. Fortunately, vacuum freeze-drying technology could address the issue of PRF storage. Freeze-drying decreases the moisture of the PRF to a minimum, and vacuum environment reduces the possibility of oxidation, denaturation and contamination (Walters et al., 2014). The two points mentioned above suggest that freeze-drying has the potential to keep the morphology of PRF and the stability of biological properties of PRF proteins, especially various growth factors (Nakajima et al., 2012). Moreover, previous study demonstrated that, compared to fresh PRF, lyophilized PRF presented better cell proliferation-promoting capacity *in vitro*, and better histocompatibility and bone regeneration *in vivo* (Li et al., 2014).

Electrospinning technology is a simple and efficient approach to acquire fibers at nanometer and micron scale (Greiner and Wendorff, 2007; Bhardwaj and Kundu, 2010). Electrospinning has the advantages of large specific surface area, high porosity, and low cost. In addition, it has been wildly studied or used in regenerative medicine, tissue engineering, medical wound dressing, and delivery of controlled drug release (Sill and von Recum, 2008). Furthermore, the ordered arrangement of nanofibers could produce complex three-dimensional (3D) multiscale and ultrathin fibrous scaffolds, and the microfabricated scaffolds could maintain cell viability better than plain scaffolds (Chen et al., 2017; Asencio et al., 2018). As new materials (such as bioactive substance) are added for preparation, electrospinning is expected to be an ideal biological scaffold. We therefore sought to further explore the biological potential of electrospinning with lyophilized PRF added as one of the materials.

Herein, we investigate the surface structure of lyophilized PRF and its effect on promoting cell proliferation and osteogenesis of MEC3T3-E1 cells. After mixing polyvinyl alcohol (PVA) and sodium alginate (SA) polymers with lyophilized PRF, we produce PVA/SA/PRF electrospinning and explore the surface morphology of the nanofibers. The effects of PVA/SA/PRF nanofibers extract on promoting cell proliferation and osteogenesis of MEC3T3-E1 cells are also investigated. Based on these results, we want to shed light on the possible strategies for tissue engineering (especially bone regeneration) or other applications with PVA/SA/PRF electrospinning.

## Materials and Methods

### Preparation of Lyophilized PRF of Mouse

The care and experimental procedures of the animals in our study were compatible with animal ethical care guideline of Xiamen Medical College, and conformed to the Guide for the Care and Use of Laboratory Animals from the National Institutes of Health. C57/BL6 mice (18–22 g) 4–6 weeks of age were anesthetized with ether, and blood was collected from eye socket. The mouse blood was centrifuged at 400 *g* for 10 min and let to sit for 5 min at room temperature, then the PRF gel layers were collected, and the blood clots at the bottom of PRF gel layers were removed. The pure PRF was frozen and stored at −80°C. The frozen PRF was then freeze-dried for 48 h at −80°C using an Alpha2-4LDplus lyophilizer (Christ, Germany). The lyophilized PRF was used in the following experiments.

### Surface Structure of Lyophilized PRF

Visualization of lyophilized PRF surface structure was achieved using a scanning electron microscope. Lyophilized PRF was fixed using 4% glutaraldehyde for 60 min. After dehydration with gradient alcohol series (30, 50, 70, 80, 90, 95, and 100%) for 15 min each, the samples were dried by hexamethyldisilazane and sputtered with gold palladium using a sputter coater. Dried lyophilized PRF samples were examined by scanning electron microscopy (SEM, ZEISS, Germany), and the diameters of 100 random pores were selected and analyzed by ImageJ (NIH, United States).

### Cell Culture of MEC3T3-E1

MEC3T3-E1 cells (preosteoblast cell line of mouse calvaria bone) were purchased from the Cell Bank of Chinese Academy of Sciences (Shanghai, China). MEC3T3-E1 cells were cultivated in Minimum Essential Medium α (MEMα, Gibco, United States) supplemented with 10% fetal bovine serum (FBS, Gibco, United States), NaHCO_3_ (1.5 g/L, Sigma, United States), inositol (43.2 mg/L, Sigma, United States), folic acid (8.82 mg/L, Sigma, United States), and β-mercaptoethanol (7.8 mg/L, Sigma, United States) in a humidified atmosphere at 37°C with 5% CO_2_. The cultivation medium was replaced every 2 days.

### Cell Proliferation Assay of Lyophilized PRF

To prepare the lyophilized PRF medium, 50 mg of lyophilized PRF was added into 3 mL of MEMα containing 100 units/mL penicillin and 100 mg/mL streptomycin. After incubation for 24 h at 4°C, the medium was centrifuged (400 *g*) for 5 min and then the supernatant was collected and filtered with a 0.22 μm filter. Then 10% FBS was added into the medium. A Cell Counting Kit-8 (CCK-8, Dojindo, Japan) was used to evaluate the proliferation ability of the MEC3T3-E1 cells cultivated in lyophilized PRF medium, and MEMα supplement with 10% FBS was set as the control medium group. A total of 3 × 10^3^ cells was seeded in each well of a 96-well plate. After cultivation in the control medium for 24 h, the medium was replaced with lyophilized PRF medium and cultivated for 1, 3, and 5 days, the cultivation medium was changed to 100 μL of control medium with 10 μL of CCK-8 solution. After being incubated for 2 h in a humidified atmosphere at 37°C with 5% CO_2_, the absorbance of the supernatant at 450 nm was examined with a spectrophotometer (Thermo, United States).

### Detection of Osteogenic Genes Expression

A total of 5 × 10^4^ cells was seeded in each well of a six-well plate. After cultivation in the control medium for 24 h, the medium was replaced with lyophilized PRF medium and cultivated for 5 days. The cells were collected for quantitative reverse transcription polymerase chain reaction (qRT-PCR) analysis. Total RNA was extracted using TriZol (Thermo, United States). The complementary DNA (cDNA) synthesis was performed using a RevertAid First Strand cDNA Synthesis Kit (Thermo, United States) on an MJ Research PTC-200 Peltier Thermal Cycler (Bio-Rad, United States). The qRT-PCR analysis was performed on a CFX96 Touch Real-Time PCR Detection System (Bio-Rad, United States). All of the procedures followed the manufacturers’ protocols and were described previously (Yang et al., 2020). We monitored the expression of osteocalcin (OCN) and osteopontin (OPN) for osteogenesis of MEC3T3-E1 cells. PCR primer sequences and PCR products sizes are listed in Table 1. Primer sequences were designed and blasted at Primer BLAST website^1^. The expression of GAPDH was used as reference for normalization. ΔΔ Ct calculation method was used to calculate and quantify the relative expression levels (Livak and Schmittgen, 2001). Three parallel replicates were prepared.

**TABLE 1:** Oligonucleotide primer sequences for RT-PCRs.

Target gene	Primer sequence (forward, reverse)
OCN	Sense 5′-ATGATGGAAGGCTCATGGTTG-3′
	Antisense 5′-TGTTGGCGTACAGGTAATAGAA-3′
OPN	Sense 5′-TCCTGGCACCTACCTAAAACAGCA-3′
	Antisense 5′-CTACACTCTCGGCATTCACTTTGG-3′
GAPDH	Sense 5′-GGTGAAGGTCGGTGTGAACG-3
	Antisense 5′-CTCGCTCCTGGAAGATGGTG-3′

### Preparation and Observation of PVA/SA/PRF Nanofibers

9 g of PVA (Sigma, United States) and 1 g of SA (Sigma, United States) were added into 90 mL of deionized water, and the PVA/SA solution was stirred at 600 revolutions per minute (rpm) for 8 h. Then 50 mg of lyophilized PRF was added into the solution. After being treated with ultrasound for 1 h, the PVA/SA/PRF solution was stirred at 600 rpm for 4 h. The PVA/SA solution without lyophilized PRF was set as control group. The PVA/SA/PRF solution or PVA/SA solution was put in 5 mL injection syringes with a 21G needle. The solutions were fabricated using an electrospinning machine at a static voltage of 20 kilovolt (kV). The distance of receiving board and flat pinhead was 25 cm, and the solution flow rate was 0.1 mL/h. The PVA/SA/PRF nanofibers and PVA/SA nanofibers were dried in a vacuum freeze drier. The surface structure of the nanofibers was observed using a scanning electron microscope, and the operating procedures were the same as mentioned in Section “Surface Structure of Lyophilized PRF.” For the measurement of the diameters of 100 random fibers, fast Fourier transform (FFT) of the SEM images and analysis of the spectral intensity distribution of the electrospinning were performed using ImageJ (NIH, United States). In particular, the spectral intensity distribution was obtained from the summation of gray value of 360 points in a 360° radial direction of the circle using ImageJ with oval profile, and the center of the circle is also the center of the FFT image.

### Cell Proliferation Assay of Nanofibers Extract

To prepare the PVA/SA/PRF and PVA/SA nanofibers extract, 50 mg of PVA/SA/PRF nanofibers or 50 mg of PVA/SA nanofibers was added into 3 mL MEMα (with 100 units/mL penicillin and 100 mg/mL streptomycin), respectively. After incubation for 24 h at 4°C, the medium was centrifuged (400 *g*) for 5 min, then the supernatant was collected and filtered with a 0.22 μm filter. Then 10% FBS was added into the medium. MEMα supplemented with 10% was set as the control medium group. CCK-8 (Dojindo, Japan) was used to evaluate the proliferation ability of the MEC3T3-E1 cells cultivated in PVA/SA/PRF and PVA/SA nanofibers extract, and the operating procedures were the same as described in Section “Cell Proliferation Assay of Lyophilized PRF.”

### Osteogenic Differentiation Assay of PVA/SA/PRF Nanofibers Extract

The osteogenesis differentiation capacity of MEC3T3-E1 influenced by PVA/SA/PRF nanofibers extract medium was evaluated by Alizarin Red S staining. A total of 1 × 10^5^ MEC3T3-E1 cells were seeded in each well of 12-well plates with PVA/SA/PRF nanofibers extract and PVA/SA nanofibers extract. After cultivation for 21 days, the supernatant was removed and washed with PBS for three times, and the cells were fixed in 4% paraformaldehyde for 30 min. After being washed with PBS for three times, the fixed cells were incubated in 0.1% Alizarin Red S solution (Sigma, United States) for 30 min, and then washed with distilled water twice. The images of the stained cells were acquired under a microscope (Olympus, Japan), and spectrophotometric analysis was performed using ImageJ (NIH, United States).

### Statistical Analysis

The data are presented as mean ± standard deviation (SD). An independent-samples *t*-test analysis of variance was used to analyze the differences between the groups. Statistical calculations were performed with IBM SPSS Statistics 26 software (IBM, United States). *P* < 0.05 is considered statistically significant.

## Results

### Morphological Characteristics of Lyophilized PRF

**FIGURE 1:**
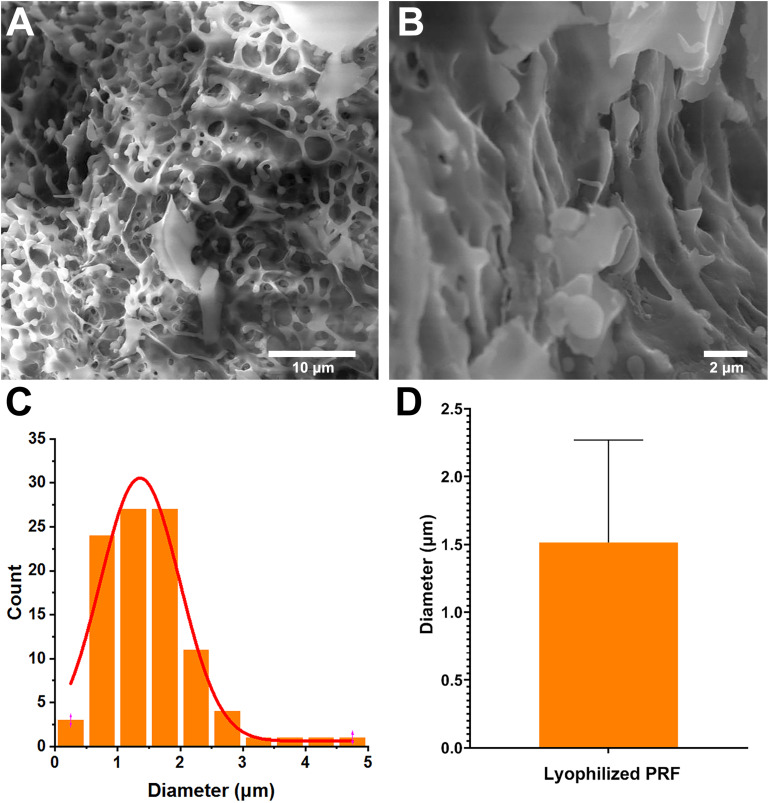
Surface parameters of lyophilized PRF. **(A)** Surface morphology of lyophilized PRF (4000×). **(B)** Surface morphology of lyophilized PRF (10,000×). **(C)** The diameter distribution of pores. **(D)** The diameters of the pores were 1.51 ± 0.75 nm.

To evaluate the surface parameters of lyophilized PRF, the surface morphology of cut samples was detected by SEM. The SEM image showed that lots of cavities were present in the lyophilized PRF (Figures 1A,B). The image analysis results revealed that the diameters of pores were mainly distributed in the range of 0.75∼1.75 μm (78%) (Figure 1C), and the distribution of the pore diameters accorded with a GaussAmp fitting curve [*y* = 0.60 + 29.93 × exp(−0.5 × ((*x*−1.36)/0.63)^2^, *R*^2^ = 0.96]. The diameters of the pores were 1.51 ± 0.75 μm (Figure 1D).

### Lyophilized PRF Promoted the Proliferation of MEC3T3-E1 Cells

**FIGURE 2:**
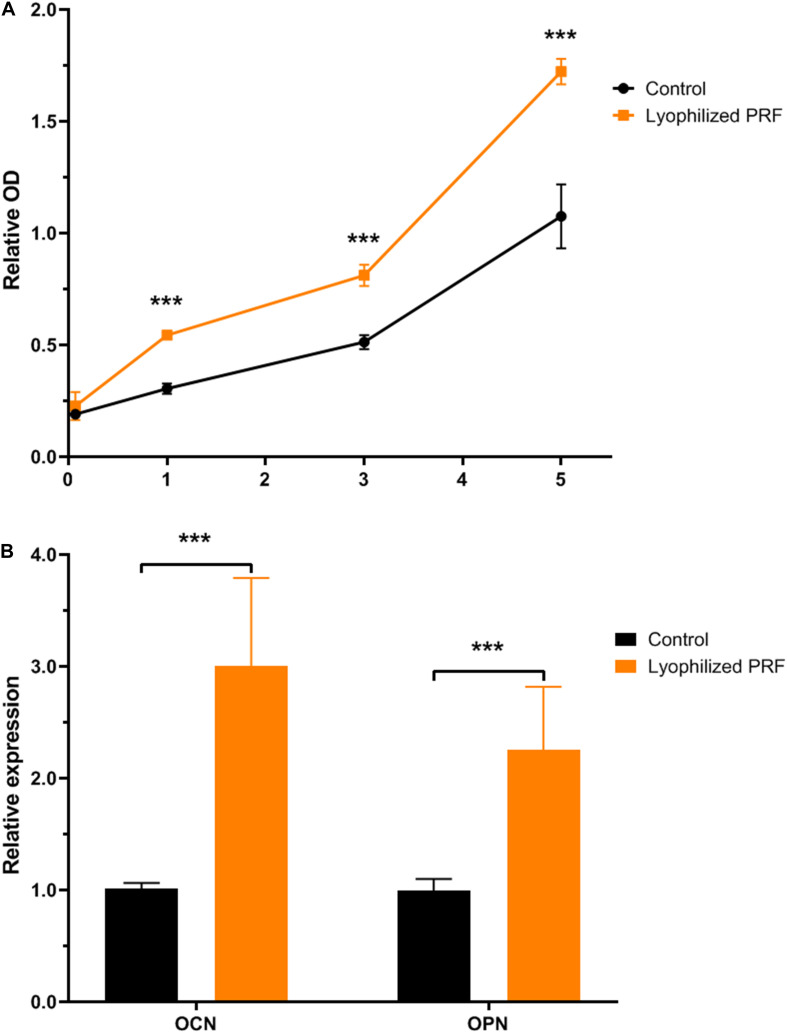
The effect of lyophilized PRF on the proliferation and differentiation potential of MEC3T3-E1 cells. **(A)** The growth kinetics of MEC3T3-E1 cells in control and lyophilized PRF medium. The result showed that lyophilized PRF medium promoted MEC3T3-E1 cells proliferation. **(B)** The differentiation potential of MEC3T3-E1 cells in control and lyophilized PRF medium. The result revealed that lyophilized PRF medium could increase the gene expressions of OCN and OPN. ***P < 0.001.

After cultivation for 1 day, the CCK-8 results revealed that lyophilized PRF medium promoted the proliferation of MEC3T3-E1 cells. After 3 days, the proliferation ability of MEC3T3-E1 cells in lyophilized PRF medium still was approximately 35% higher than that in control medium. The CCK-8 results on the fifth day showed that the excellent potential of the proliferation ability was kept to the end of experimental time point (Figure 2A).

### Lyophilized PRF Increased the Gene Expressions of OCN and OPN

The qRT-PCR assay was used to determine the effect of lyophilized PRF on osteogenic differentiation of MEC3T3-E1 cells, and the results revealed that the expression level of OCN in PRF medium was threefold higher than that in control group. Meanwhile, OPN expression in PRF medium was also approximately twofold higher than that in control group (Figure 2B).

### Surface Features of PVA/SA/PRF Nanofibers

**FIGURE 3:**
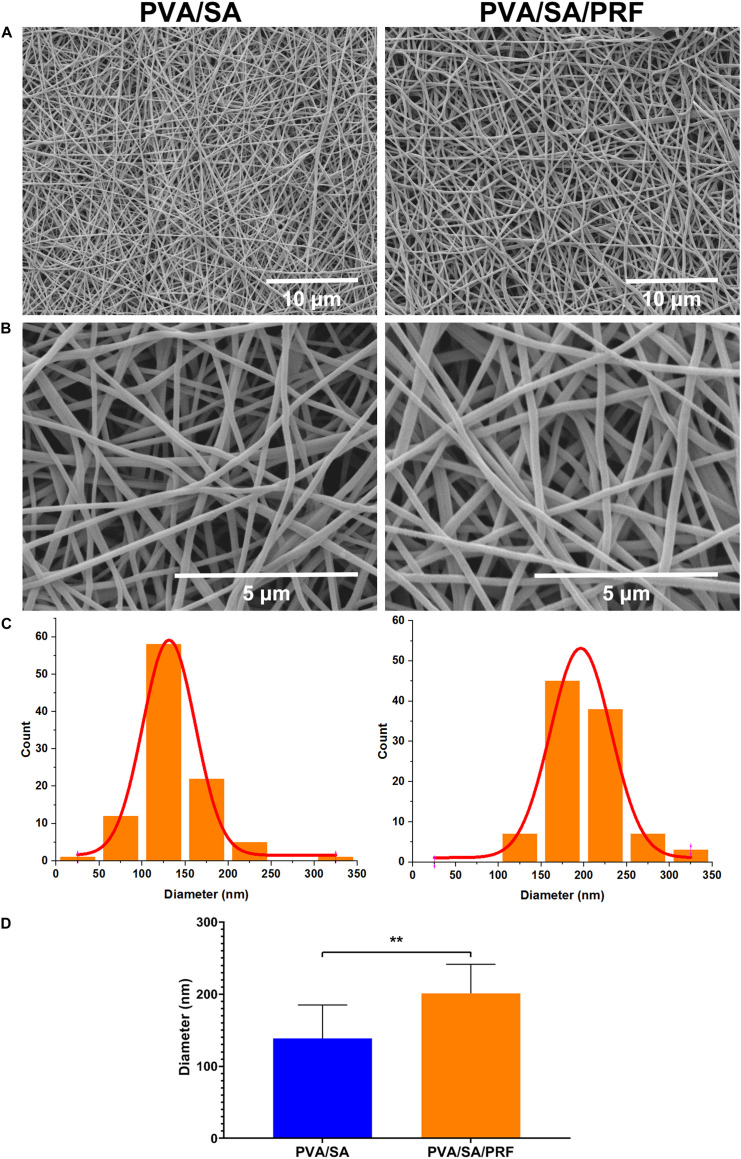
Surface features of nanofibers of PVA/SA and PVA/SA/PRF. **(A)** Surface morphology of nanofibers of PVA/SA and PVA/SA/PRF (6000×). **(B)** Surface morphology of nanofibers of PVA/SA and PVA/SA/PRF (24,000×). **(C)** The diameter distribution of PVA/SA and PVA/SA/PRF fibers. **(D)** The diameters of the PVA/SA fibers and PVA/SA/PRF fibers were 138.66 ± 46.20 and 201.14 ± 40.14 nm, respectively. **P < 0.01.

**FIGURE 4:**
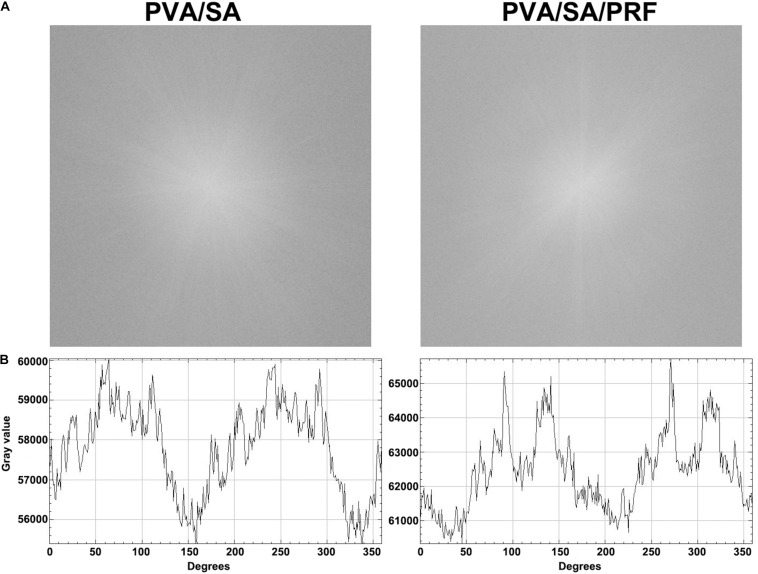
The FFT image and spectral intensity distribution of PVA/SA and PVA/SA/PRF nanofibers. **(A)** The FFT was performed for PVA/SA and PVA/SA/PRF nanofibers. **(B)** The spectral intensity distribution demonstrated the diversity between PVA/SA/PRF fibers and PVA/SA fibers.

Scanning electron microscopy assay was also used to determine the surface parameters of PVA/SA and PVA/SA/PRF nanofibers. The SEM image showed that the fibers of PVA/SA/PRF were lager than PVA/SA fibers (Figures 3A,B). The image analysis results revealed that the diameters of PVA/SA fibers were mainly distributed in the range of 100∼150 nm (58%), and the distribution of the fiber diameters accorded with a GaussAmp fitting curve [*y* = 1.52 + 57.60 × exp(−0.5 × ((*x*−131.26)/30.58)^2^, *R*^2^ = 0.99]. While the diameters of PVA/SA/PRF fibers were mainly distributed in the range of 150∼250 nm (83%), and the distribution of the fiber diameters accorded with a GaussAmp fitting curve [*y* = 1.09 + 52.04 × exp(−0.5 × ((*x*−196.29)/35.41)^2^, *R*^2^ = 0.99] (Figure 3C). The diameters of the PVA/SA fibers and PVA/SA/PRF fibers were 138.66 ± 46.20 vs. 201.14 ± 40.14 nm (Figure 3D). The FFT image showed a large difference between the gray value distribution of PVA/SA/PRF fibers and that of PVA/SA fibers (Figure 4A). The radial sums of PVA/SA FFT image showed that the gray value of spectral intensity distribution ranged from 55,400 to about 60,000, while the gray value of spectral intensity distribution of PVA/SA/PRF ranged from 60,400 to about 65,700 (Figure 4B). The spectral intensity distribution at 360° radius direction also demonstrated the diversity between PVA/SA/PRF fibers and PVA/SA fibers.

### PVA/SA/PRF Nanofibers Extract Promoted the Proliferation of MEC3T3-E1 Cells

**FIGURE 5:**
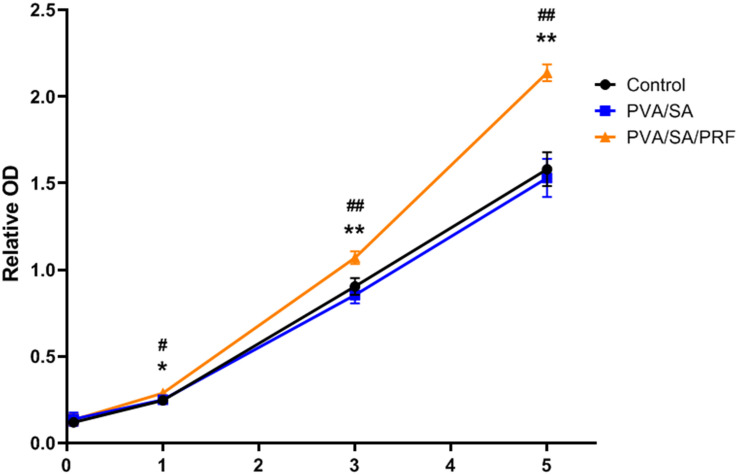
The effect of PVA/SA and PVA/SA/PRF nanofibers extract on the proliferation ability of MEC3T3-E1 cells. PVA/SA/PRF nanofibers extract promoted the proliferation ability of MEC3T3-E1 cells obviously. *P < 0.05 (PVA/SA/PRF vs. Control); **P < 0.01 (PVA/SA/PRF vs. Control); ^#^*P* < 0.05 (PVA/SA/PRF vs. PVA/SA); ^##^*P* < 0.01 (PVA/SA/PRF vs. PVA/SA).

Cell Counting Kit-8 assay was also used to evaluate the proliferation ability of MEC3T3-E1 cells and the cytotoxicity of the PVA/SA and PVA/SA/PRF nanofibers extract. Compared with PVA/SA nanofibers extract and control groups, PVA/SA/PRF nanofibers extract promoted the proliferation ability of MEC3T3-E1 cells obviously on the first, third, and fifth day of the experimental time points. Meanwhile, there was no significant difference on the proliferation of MEC3T3-E1 cells between PVA/SA nanofibers extract and control medium in the duration of 7 days (Figure 5).

### PVA/SA/PRF Nanofibers Extract Enhanced Osteogenic Differentiation

**FIGURE 6:**
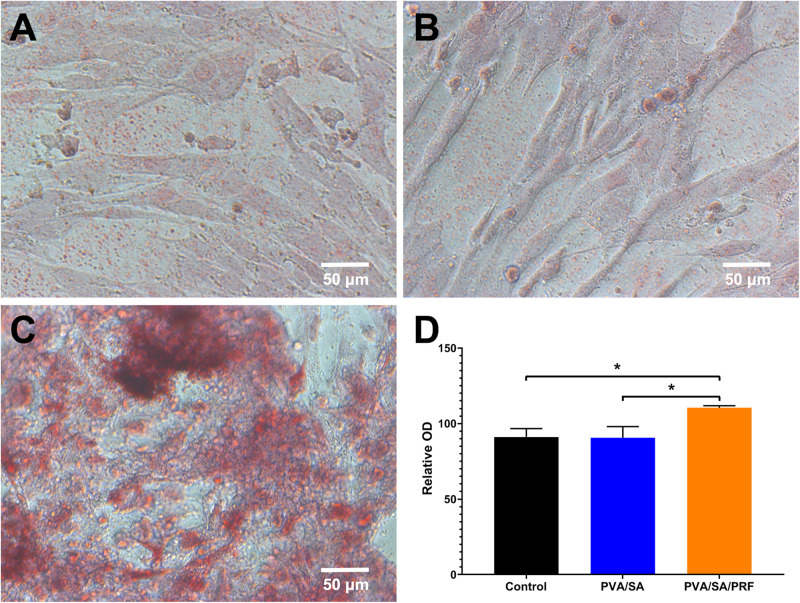
The effect of PVA/SA and PVA/SA/PRF nanofibers extract on the osteogenic differentiation of MEC3T3-E1 cells. **(A)** Osteogenic differentiation of MEC3T3-E1 cells in control medium. **(B)** Osteogenic differentiation of MEC3T3-E1 cells in PVA/SA nanofibers extract. **(C)** Osteogenic differentiation of MEC3T3-E1 cells in PVA/SA/PRF nanofibers extract, and many stained calcified nodules could be observed in this group. **(D)** The spectrophotometric analysis results of the Alizarin Red S staining images. *P < 0.5.

Alizarin Red S was used to stain the calcified nodules accumulated by osteogenic differentiation of MEC3T3-E1 cells. The results revealed that, compared with PVA/SA nanofibers extract and control groups, the relative optical density (OD) in PVA/SA/PRF nanofibers extract increased significantly. However, compared with control group, PVA/SA electrospinning extract medium did not enhance osteogenic differentiation of MEC3T3-E1 cells (Figure 6).

## Discussion

Platelet-rich fibrin and electrospinning technology are promising therapeutic intervention in tissue engineering and have been widely studied (Oryan et al., 2016; Dohle et al., 2018; Nemati et al., 2019; Udomluck et al., 2019). Bone tissue engineering is an alternative therapeutic strategy to repair damaged bone (Nemati et al., 2019; Udomluck et al., 2019). The purpose of this study was to investigate the biological characteristics of lyophilized PRF and its electrospinning, and explore the potential of PVA/SA nanofibers with lyophilized PRF added as the biomaterial for bone tissue engineering. The data from the present study demonstrated that both lyophilized PRF and PVA/SA/PRF nanofibers could promote the proliferation and mineralization activity of MEC3T3-E1 cells.

Platelet-rich plasma is plasma with a higher concentration of platelets, and has been used for wound healing and tissue engineering (Andreone and den Hollander, 2019). To overcome the disadvantages such as coagulopathies and allergic reactions after addition of bovine thrombin, PRF as the second generation of platelet products was developed. In fact, previous report demonstrated that PRF presents better capacity than PRP to alleviate the negative effect of drugs on the proliferation, migration, and viability of osteoblasts and oral fibroblasts as expect (Steller et al., 2019). However, PRF also has several limitations, not the least of which is that it requires the preparation for immediate use (Morris, 2005; Choukroun et al., 2006). Fortunately, vacuum freeze-drying technology addressed the issue of PRF storage and could maintain or even enhance the biological functions of PRF (Pietramaggiori et al., 2006; Nakajima et al., 2012; Li et al., 2014). It was speculated that the enhancement of biological function was due to the enlargement of pore size and improvement of cytokines release (Li et al., 2014). In addition, our data demonstrated that the diameters of the pores of our lyophilized PRF from mouse were 1.51 ± 0.75 μm, which are smaller than the 8.06 ± 0.31 μm of lyophilized PRF but larger than 0.6 ± 0.13 μm of fresh PRF from pig in a previous study (Li et al., 2014). The results suggested that the species of the source might influence the pore size of lyophilized PRF.

The present study indicated that lyophilized PRF promoted the proliferation and increased the OCN and OPN gene expressions of MEC3T3-E1 cells when compared to MEMα supplement with 10% FBS, suggesting that lyophilized PRF contains more nutriments than FBS for MEC3T3-E1 cells. These biological functions to promote proliferation and mineralization were attributed to the ability of the vacuum freeze-drying technology to maintain the bioactivity of the cytokines including abundant growth factors in the PRF. Growth factors play a critical role in tissue engineering (Udomluck et al., 2019). As one of the three elements for tissue engineering, many growth factors are well-studied including bone morphogenetic proteins (BMP2 to BMP8), TGF-β, PDGF, FGF, and so on (Gittens and Uludag, 2001; De Witte et al., 2018; Kim et al., 2018). In particular, bFGF, also known as FGF-2, has been used for the clinical regenerative treatment of osteonecrosis of the femoral head, demonstrating its safety and efficacy (Kuroda et al., 2019). It is interesting that plenty of growth factors for bone tissue engineering were involved in the cytokine profiles of PRF (Masuki et al., 2016). The growth factors in lyophilized PRF might assist each other in strengthening the mineralization activity of MEC3T3-E1 cells.

Electrospinning technology provides a potential and efficient platform to acquire nanofibers used as artificial biological scaffold for bone tissue engineering (Greiner and Wendorff, 2007; Bhardwaj and Kundu, 2010; Udomluck et al., 2019). Electrospinning has the advantages of large specific surface area, high porosity, and low cost, which meets the requirements of bone tissue scaffold to integrate a mass of bone related cells for forming bone like tissues (Yoshimoto et al., 2003; Udomluck et al., 2019). Moreover, growth factors were added for preparation, and the growth factors could be sustainably released from the electrospinning nanofibers (Sahoo et al., 2010). Herein, we intended to combine both the advantages of the lyophilized PRF and electrospinning technology to mimic the nature of the extracellular matrix. We speculated that while lyophilized PRF was added for electrospinning nanofibers preparation, plenty of the growth factors could be released from the electrospinning and benefit bone tissue formation. Our data demonstrated that the diameters of the traditional PVA/SA fibers were 138.66 ± 46.20 nm, but after lyophilized PRF being added, the diameters of the fibers were enlarged to 201.14 ± 40.14 nm. The FFT analysis also showed that gray value of spectral intensity distribution range went from 55,400∼60,000 to 60,400∼65,700. These results indicated that lyophilized PRF added as a component of the electrospinning material modified the physical properties of the nanofibers, especially the surface characteristics, which might be important for bone tissue engineering.

Previous studies focused on surface modification for enhancing cellular behavior in tissue engineering (high proliferation rate or bone formation), which indicated that proliferation and mineralization activity of the bone related cells is important for bone tissue engineering (Nandakumar et al., 2013; Lee et al., 2016; Udomluck et al., 2019). Electrospinning nanofibers with sustained release of growth factors have the great potential to create a beneficial microenvironment for bone tissue formation, including promoting migration and differentiation of osteoblasts by angiogenic, inflammatory, and bone formation enhancement (Sui et al., 2007; Vo et al., 2012). It is interesting that the cytokines from PRF also have similar biological functions for tissue regenerative medicine, such as anti-inflammation effects, promoting osteogenic differentiation and vascularization (Pietramaggiori et al., 2006; You et al., 2019; Nasirzade et al., 2020; Xie et al., 2020; Zhang et al., 2020). In present study, we investigated the effect of extract of PVA/SA/PRF nanofibers on proliferation and osteogenesis of osteogenic precursor cells (MEC3T3-E1 cells) *in vitro*. The results showed that PVA/SA/PRF nanofibers extract presented an extraordinary capacity to promote the proliferation and mineralization activity of MEC3T3-E1 cells in contrast to PVA/SA nanofibers extract and control medium, which demonstrated that PVA/SA/PRF nanofibers could maintain the bioactivity of proliferation and osteogenesis-promoting cytokines, and release them to the medium.

## Conclusion

This study investigated the biological properties of lyophilized PRF, as promoting MEC3T3-E1 cells proliferation and mineralization induction. In addition, lyophilized PRF added into PVA/SA polymers as a component for electrospinning preparation could modify the physical properties of the electrospinning nanofibers. Moreover, PVA/SA/PRF nanofibers extract also promoted the proliferation and mineralization activity of MEC3T3-E1 cells in contrast to PVA/SA nanofibers extract and control medium. These results indicated that PVA/SA/PRF nanofibers might maintain the characteristics of lyophilized PRF for enhancing proliferation and mineralization, which might be a candidate scaffold for bone tissue engineering.

## Data Availability Statement

The original contributions presented in the study are included in the article/supplementary material. Further inquiries can be directed to the corresponding author/s.

## Ethics Statement

The animal study was reviewed and approved by the Ethics Committee of Stomatological Hospital of Xiamen Medical College.

## Author Contributions

PW performed the 1 assay. JN, YS, and SMZ performed the 2–8 assay. YJS gathered and analyzed the research data. JN conceptualized and designed the study, wrote the manuscript, and reviewed and revised the final manuscript. YL critically reviewed and revised the manuscript for important intellectual content. All authors contributed for the development of this manuscript, approved the final version of the manuscript as submitted, and agreed to be accountable for all aspects of the work in ensuring that questions related to the accuracy or integrity of any part of the work.

## Conflict of Interest

The authors declare that the research was conducted in the absence of any commercial or financial relationships that could be construed as a potential conflict of interest.

## Footnotes

1 https://www.ncbi.nlm.nih.gov/tools/primer-blast/index.cgi
